# An Acoustofluidic Capillary Nozzle for Programmable Microstructure Assembly in Direct Ink Writing of Flexible Conductive Composites

**DOI:** 10.3390/mi17060744

**Published:** 2026-06-20

**Authors:** Minghao Shao, Chaohui Wang, Tengfei Zheng, Jiahe Liang

**Affiliations:** 1 State Key Laboratory for Manufacturing Systems Engineering, Xi’an Jiaotong University, Xi’an 710049, China; shaominghao123@stu.xjtu.edu.cn (M.S.); chhw@xjtu.edu.cn (C.W.); 2 Shaanxi Key Lab of Intelligent Robots, Xi’an Jiaotong University, Xi’an 710049, China; 3 3D Printing Research Center, Department of Ultrasonic Medicine, Tang Du Hospital, Air Force Medical University, No. 569 of Xin Si Road, Xi’an 710038, China

**Keywords:** acoustofluidic capillary nozzle, direct ink writing, programmable microstructure assembly, filler particle alignment, conductive composites

## Abstract

The spatial organization of microscale fillers is critical for macroscopic performance, yet precise control over their distribution and orientation remains a major challenge in direct ink writing. Here, we present an acoustofluidic capillary nozzle that integrates acoustic manipulation into direct ink writing, enabling programmable in situ assembly of functional fillers during extrusion. By coupling a piezoelectric transducer with a commercial glass capillary, stable acoustic standing waves are established within the flow channel, driving suspended filler particles toward pressure nodes via acoustic radiation forces. Simulations and experiments systematically investigate how capillary geometry and material properties influence acoustic energy distribution and particle assembly behavior. In particular, rectangular capillaries generate stable multi-node standing waves, inducing periodic alignment of nickel-coated carbon fibers into ordered conductive bundles. This acoustically programmed microstructure reduces the percolation threshold from 8 wt% to 2 wt% and enhances electrical conductivity by up to 32.1-fold at identical filler contents. Meanwhile, the composites exhibit pronounced anisotropic conductivity and maintain excellent mechanical flexibility, with stable electromechanical performance under 16% bending strain and cyclic loading. This work demonstrates a simple and scalable acoustofluidic nozzle platform for programmable microstructure engineering in direct ink writing, offering new opportunities for fabricating high-performance multifunctional composites.

## 1. Introduction

The spatial organization of micro- and nanoscale building blocks plays a decisive role in determining the macroscopic properties and functionalities of advanced composites [1,2,3]. In biological systems, the alignment and arrangement of cells critically regulate tissue functions, such as neuron regeneration [4,5], cardiac contraction [6,7], and tendon mechanics [8,9]. Similarly, in engineered materials, the orientation and spatial distribution of functional fillers strongly influence electrical conductivity [10,11], mechanical anisotropy [12,13], and structural integrity [14,15]. Inspired by these biological and engineered systems, precise control over microstructural organization has become one of the central objectives in the design of high-performance multifunctional materials.

Direct ink writing (DIW), as a versatile extrusion-based additive manufacturing technique, has attracted extensive attention for fabricating complex three-dimensional architectures in functional composite systems [16,17,18], soft robotics [19,20], and tissue engineering [21,22,23]. Despite its advantages in geometric freedom and material compatibility, a fundamental limitation of DIW lies in its inability to actively regulate the spatial distribution and orientation of functional fillers during the extrusion process. In conventional DIW, filler organization is largely governed by passive effects such as shear-induced alignment or random dispersion, which are difficult to control and often lead to heterogeneous microstructures. Consequently, the intrinsic structure–property relationship in composite materials cannot be fully exploited, thereby limiting the functional performance of printed components.

To address this challenge, external-field-assisted strategies have been explored to actively manipulate filler distribution and orientation during material processing. Magnetic fields [24], electric fields [25,26], and hydrodynamic forces [27] have shown the ability to guide microscale objects and tune filler organization. Nevertheless, these methods often suffer from material selectivity, complex system integration, limited scalability, or relatively low throughput [28,29,30]. Acoustic microfluidics, also known as acoustofluidics, provides a particularly attractive alternative for contactless and label-free manipulation of particles, droplets, cells, and fibers in fluidic environments [31]. Acoustic radiation forces generated by standing acoustic waves can drive suspended objects toward pressure nodes or antinodes, depending on their acoustic contrast with the surrounding medium [32]. Compared with magnetic or electric manipulation, acoustofluidic manipulation offers advantages including material universality, biocompatibility, simple actuation, and compatibility with continuous-flow systems [29,33,34,35]. These characteristics make acoustofluidics highly promising for integration with additive manufacturing processes, where real-time and in situ microstructure regulation is desired.

Recent studies have demonstrated the feasibility of integrating acoustic fields with extrusion-based 3D printing to manipulate particles, cells, or functional fillers during deposition and to fabricate structured materials [29,36,37,38]. In precision mechatronic systems, stable signal control and phase adjustment are important for improving system repeatability and measurement accuracy, as demonstrated in high-precision electro-hydraulic displacement measurement [39]. Similarly, stable excitation control is essential in acoustofluidic printing systems to generate reproducible acoustic fields and achieve programmable microstructure assembly. However, many existing acoustic-assisted printing studies mainly focus on demonstrating particle manipulation or pattern formation, while the influence of nozzle geometry and material properties on in-nozzle acoustic-field distribution, pressure-node formation, and focusing efficiency has not been systematically clarified. These gaps hinder the rational design of simple, scalable, and DIW-compatible acoustofluidic nozzles for programmable microstructure engineering.

In this work, we propose an acoustofluidic capillary nozzle for programmable in situ assembly of functional fillers during direct ink writing. By directly coupling a piezoelectric transducer (PZT) with a commercial glass capillary, the printing nozzle is transformed into an active acoustic resonator without complex microfabrication or structural modification. Stable standing acoustic waves are generated within the capillary channel, enabling suspended fillers to migrate toward pressure nodes and assemble into ordered microstructures during extrusion. The main contributions of this work are twofold. First, numerical simulations and experimental observations are combined to systematically investigate how capillary geometry and material properties influence acoustic energy distribution, pressure-node formation, and particle focusing efficiency. Second, the acoustofluidic nozzle is further applied to the in situ assembly of nickel-coated carbon fibers within PEGDA-based ink, demonstrating the construction of patterned conductive networks at low filler loadings. The nozzle is based on commercially available glass capillaries and simple PZT integration, providing a DIW-compatible and scalable platform for programmable microstructure engineering in conductive composites. This study advances acoustic-assisted DIW from model particle manipulation toward design-guided nozzle engineering and functional conductive composite fabrication.

## 2. Materials and Methods

### 2.1. Acoustofluidic Capillary Nozzle and Filler Manipulation Mechanism

**Figure 1:**
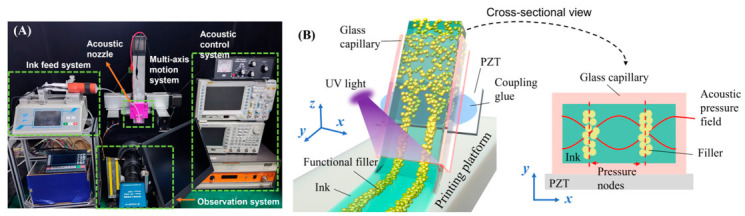
Schematic illustration of the DIW system integrated with an acoustofluidic capillary nozzle and its acoustic manipulation mechanism. (**A**) Photograph of the DIW setup, consisting of the acoustofluidic capillary nozzle, a multi-axis motion system, an ink delivery system, an acoustic control system, and an observation system. (**B**) Schematic of particle manipulation within the acoustofluidic capillary nozzle under acoustic excitation. The inset shows the cross-sectional view, where a standing acoustic pressure field is established, driving filler particles toward pressure nodes via acoustic radiation forces, resulting in the formation of periodically aligned particle bundles during extrusion.

Figure 1 illustrates the configuration of the custom-built acoustofluidic DIW system and the corresponding particle manipulation mechanism. As shown in Figure 1A, the system consists of an acoustofluidic capillary nozzle, a multi-axis motion system, an ink delivery system, an acoustic control system, and an observation and imaging system. Detailed system parameters, including the motion range, positioning accuracy, hardware configuration, and nozzle assembly scheme, are provided in Section S1 of the Supplementary Materials. The acoustofluidic capillary nozzle was constructed by directly bonding a piezoelectric transducer (Risun Electronic, Kunshan, China) onto a commercial glass capillary (ALFAQUARTZ, Lianyungang, China) through an epoxy resin (Bob Smith Industries, Atascadero, CA, USA) coupling layer. Under sinusoidal electrical excitation, the PZT generates high-frequency mechanical vibrations, which are transmitted into the fluid inside the capillary channel through the capillary wall. As a result, stable acoustic standing waves are established within the flow channel.

During printing, the ink was extruded through the nozzle while the acoustically induced filler organization was fixed by means of rapid UV curing immediately after filament deposition, enabling in situ microstructure assembly during DIW. Figure 1B schematically illustrates the acoustofluidic manipulation process and the corresponding acoustic field distribution within the capillary cross-section. Under acoustic excitation, suspended particles or fillers migrate from an initially random distribution toward pressure nodes under acoustic radiation forces and undergo spatially confined aggregation during flow. This process results in periodically aligned stripe-like structures within the extruded filament. In contrast, without acoustic excitation, particles are passively transported by the fluid and remain randomly distributed. Therefore, the acoustofluidic capillary nozzle functions not only as a material extrusion outlet but also as an active acoustic resonator for programmable filler assembly.

When a standing acoustic wave is established within the acoustofluidic capillary nozzle, suspended fillers are primarily subjected to acoustic radiation forces ($F_{rad}$) and viscous drag forces ($F_{drag}$). The acoustic radiation force arises from the scattering of acoustic waves by the particles and drives them toward pressure nodes or antinodes depending on their acoustic contrast factor, whereas the viscous drag force is associated with the relative motion between particles and the surrounding fluid [40,41,42]. By tuning the magnitude and direction of these forces, precise manipulation of particles can be achieved. The acoustic radiation force drives particles toward pressure nodes and depends on both particle properties and the surrounding medium. For particles much smaller than the acoustic wavelength, the force can be described by the gradient of the Gor’kov potential $U_{rad}$ [41,43]:

(1) $$F_{rad}=-\nabla U_{rad}$$

(2) $$U_{rad}=\frac{4}{3}\pi r^{3}\left(\frac{1}{2}f_{1}\frac{1}{c_{0}^{2}\rho_{0}}\langle p_{1}^{2}\rangle -\frac{3}{4}f_{2}\rho_{0}\langle v_{1}\cdot v_{1}\rangle \right)$$

(3) $$f_{1}=1-\frac{K_{p}}{K_{0}},f_{2}=\frac{2\left(\rho_{p}-\rho_{0}\right)}{2\rho_{p}+\rho_{0}}$$

where $K_{p}$ and $K_{0}$ denote the compressibility of the particle and fluid, respectively; r and $\rho_{p}$ represent the particle radius and density; $\rho_{0}$ denotes the fluid density; $f_{1}$ and $f_{2}$ are the dimensionless scattering coefficients for monopole and dipole, respectively. Here, $p_{1}$ and $v_{1}$ denote the first-order acoustic pressure and velocity fields, respectively. Symbols in bold refer to vector quantities, < > denotes time averaging. The direction and magnitude of the acoustic radiation force are closely related to the acoustic contrast factor ϕ, which can be expressed as follows [44,45]:

(4) $$\phi \left(\kappa ,\rho \right)=\frac{1}{3}f_{1}+\frac{1}{2}f_{2}=\frac{1}{3}\left(\frac{5\rho_{p}-2\rho_{0}}{2\rho_{p}+\rho_{0}}-\frac{\kappa_{p}}{\kappa_{0}}\right)$$

A larger absolute value of ϕ generally indicates a stronger acoustic radiation force under the same acoustic-field condition, leading to faster migration of suspended fillers toward the stable acoustic trapping positions. In the present nozzle, fillers with positive acoustic contrast migrate toward the pressure nodes formed in the standing acoustic field.

In addition, due to the relative motion between particles and the fluid, a viscous drag force acts on the particles, which can be expressed as follows [41]:

(5) $$F_{drag}=6\pi \mu a_{c}\left(v_{str}-v_{prt}\right)$$

where μ is the dynamic viscosity of the fluid, $v_{prt}$ is the particle velocity, and $v_{str}$ is the acoustic streaming velocity.

During ink extrusion, acoustic focusing is governed by the competition among acoustic radiation force, viscous drag, and flow-induced disturbance within the finite residence time of the ink in the acoustic actuation region. As indicated by Equation (5), an increase in fluid viscosity directly enhances the viscous drag acting on suspended fillers, thereby reducing their migration velocity toward pressure nodes under a given acoustic radiation force. Therefore, the acceptable viscosity range and allowable printing speed are not fixed values, but depend on acoustic intensity, filler size and acoustic contrast, flow rate, nozzle geometry, and curing rate. Effective focusing requires that fillers migrate to the pressure nodes within the available residence time before exiting the nozzle; otherwise, incomplete alignment and reduced microstructural fidelity may occur.

In addition, filler size determines whether acoustic radiation force or acoustic-streaming-induced drag dominates the particle motion. Previous studies have shown that, by equating these two forces, a critical particle diameter can be estimated to distinguish the radiation-force-dominated and streaming-drag-dominated regimes. For example, a critical particle diameter of approximately 1.6 μm was reported for single-particle motion in a half-wavelength resonance, above which particle motion is mainly dominated by acoustic radiation force, whereas smaller particles are more strongly affected by acoustic streaming-induced drag [46,47]. Since the acoustofluidic nozzle relies on acoustic radiation forces to drive suspended fillers toward pressure nodes, effective acoustic focusing requires that the fillers possess a sufficient acoustic contrast with the surrounding medium and that their equivalent size exceed the critical diameter for radiation-force-dominated motion.

### 2.2. Numerical Modeling of Acoustic Fields in Capillary Nozzles

To systematically investigate the effects of capillary geometry and material properties on acoustic field distributions, numerical simulations were performed using the finite element software COMSOL Multiphysics 6.0. A two-dimensional cross-sectional model was adopted to reduce computational cost while capturing the dominant transverse acoustic field characteristics within the capillary channel. This simplified model was used primarily to compare the effects of capillary geometry and wall material on cross-sectional pressure-node distributions and acoustic energy density under identical excitation conditions. Since particle and fiber migration in this work mainly occurs across the channel width toward pressure nodes, the transverse standing-wave field provides the most relevant information for evaluating acoustic focusing behavior.

It should be noted that the actual acoustofluidic nozzle is a three-dimensional coupled system involving the PZT actuator, epoxy resin coupling layer, capillary wall, and fluid domain. Therefore, axial vibration modes, inlet/outlet boundary conditions, acoustic attenuation along the printing direction, and the finite dimensions of the PZT actuator may influence the local acoustic intensity and spatial uniformity of the standing-wave field. These three-dimensional effects are not fully resolved in the present two-dimensional model. Nevertheless, the model captures the main transverse resonance characteristics, and the experimentally observed particle focusing patterns and node spacings at different excitation frequencies show good agreement with the numerical predictions, supporting the use of this simplified model for comparative nozzle design.

Two representative capillary geometries, namely circular and rectangular cross-sections, were considered. The circular capillary had a diameter of 2 mm, while the rectangular capillary had dimensions of 5.0 × 1.8 mm^2^. These dimensions were selected to ensure that the cross-sectional areas of the two geometries were of the same order of magnitude, thereby enabling a fair comparison of acoustic field characteristics. To evaluate the influence of material properties on acoustic energy transmission, three typical materials—glass, polymethyl methacrylate (PMMA), and steel—were used for the capillary walls.

In the multiphysics setup, the pressure acoustics module was employed to model acoustic wave propagation in the fluid domain, while the Solid Mechanics module was used to describe structural vibrations of the capillary. The excitation of the piezoelectric transducer (Risun Electronic, Kunshan, China) was approximated by applying a harmonic displacement excitation boundary condition at the capillary wall. The displacement amplitude is closely related to the material properties of the piezoelectric transducer and the excitation voltage (see Section S2 in the Supplementary Materials for details [48,49,50]). In this study, the displacement amplitude of the selected PZT-43 shows a linear correlation with the excitation voltage. Accordingly, the applied displacement amplitude is an estimate obtained from the piezoelectric-structural analysis performed on the PZT transducer. In addition, For the rectangular capillary, the harmonic displacement was applied to the flat wall bonded to the PZT, because this wall can be effectively coupled to the planar PZT surface through the epoxy resin coupling layer. For the circular capillary, only a limited arc region was directly coupled to the planar PZT; therefore, the harmonic displacement was applied to approximately one-quarter of the circular wall.

At the fluid–solid interface, acoustic reflection and structural vibration coupling were considered to describe acoustic confinement within the capillary channel. Material properties, including density, speed of sound, Young’s modulus, and Poisson’s ratio, were assigned according to Table 1. To ensure comparability, all models were simulated under identical geometric scales and excitation conditions. To quantitatively evaluate the acoustic field strength, the time-averaged acoustic energy density was adopted as a key metric. This parameter represents the total acoustic energy stored per unit volume and accounts for both potential and kinetic energy contributions. It is defined as follows [51]:

(6) $$\bar{E_{ac}}=\frac{1}{V}\int_{V}\left(\frac{1}{2\rho_{0}c_{0}^{2}}\langle p_{1}^{2}\rangle +\frac{1}{2}\rho_{0}\langle v_{1}\cdot v_{1}\rangle \right)dV$$

where $p_{1}$ and $v_{1}$ denote the first-order acoustic pressure and particle velocity, respectively, and < > indicates time averaging.

By sweeping the excitation frequency and calculating the corresponding acoustic energy density, resonance frequencies can be identified as the frequencies at which the energy density reaches its maximum. Furthermore, this metric enables quantitative comparison of acoustic field intensity across different geometries and material configurations. Since the acoustic radiation force is closely related to the gradient of acoustic energy density, higher energy density generally corresponds to stronger particle manipulation capability. Therefore, combining acoustic pressure distributions with energy density analysis provides a comprehensive framework for evaluating the performance of acoustofluidic capillary nozzles in particle focusing and microstructure formation.

### 2.3. Experimental Characterization of Acoustofluidic Particle Focusing

To evaluate the particle focusing performance of acoustofluidic capillary nozzles with different geometries, an in situ observation platform based on bulk acoustic wave excitation was established. The acoustofluidic capillary nozzle was driven by a sinusoidal signal generated by a function generator (AFG3251C, Tektronix, Beaverton, OR, USA), which was amplified using a power amplifier (BA4850, NF Corporation, Yokohama, Japan) and applied to a piezoelectric transducer (P-43, Risun Electronic, Kunshan, China). The transducer was bonded to a glass capillary (ALFAQUARTZ, Lianyungang, China) using an epoxy resin (Bob Smith Industries, Atascadero, CA, USA) coupling layer to ensure efficient acoustic coupling. A microscopic imaging system (Eclipse LV100, Nikon Corporation, Tokyo, Japan) was employed to monitor the real-time motion of particles within the microchannel. Polystyrene (PS) microspheres with a diameter of 10 μm were used as model particles and dispersed in deionized water to form a dilute suspension. The suspension was injected into the capillary nozzle at a constant flow rate using a syringe pump. Particle focusing behavior under different acoustic excitation conditions was systematically investigated. All experiments were conducted under identical flow rates and particle concentrations to ensure comparability.

To capture the three-dimensional migration and assembly behavior of particles, a right-angle prism was positioned adjacent to the nozzle, enabling simultaneous front-view and side-view imaging. The acquired images were processed using ImageJ software (version 1.53t, National Institutes of Health, Bethesda, MD, USA). Grayscale intensity profiles along the cross-sectional direction were extracted to quantify the focusing width and spatial periodicity of particle bands. In addition, the time required for particles to evolve from a random distribution to a stable patterned structure was measured to evaluate focusing efficiency. Key parameters, including band width, node spacing, and positional deviation, were further extracted for quantitative comparison of acoustic manipulation performance.

### 2.4. Fabrication and Characterization of Acoustically Patterned Conductive Composites

Conductive composites were fabricated using the acoustofluidic capillary nozzle developed in this study by dispersing nickel-coated carbon fibers (Ni–CF; Carbonene Technology, Shenzhen, China; average diameter d ≈ 7.5 μm and length l ≈ 100 μm) into a polyethylene glycol diacrylate (PEGDA400, Macklin Biochemical Co., Shanghai, China) matrix. To ensure uniform dispersion of Ni–CF within the resin, a pre-dispersion process was carried out prior to printing. Specifically, the weighed Ni–CF was first ultrasonically treated in anhydrous ethanol for 15 min to break up agglomerates and remove surface-adhered air bubbles. Subsequently, PEGDA400 was gradually added dropwise under mechanical stirring (S312-90 W, Zhenhe Instruments, Hangzhou, China), together with 0.5 wt% of a nonionic dispersant (Pluronic F127, Aladdin, Shanghai, China) to enhance fiber wettability and dispersion stability. Finally, 1.0 wt% of photoinitiator TPO (diphenyl (2,4,6-trimethylbenzoyl) phosphine oxide) was introduced, followed by low-speed stirring for 5 min. The mixture was then degassed to remove residual air bubbles and large agglomerates. The acoustofluidic system employs an in situ post-deposition UV curing strategy, in which the extruded filaments are exposed to UV irradiation immediately after leaving the acoustofluidic nozzle. This rapid curing process effectively immobilizes the acoustically aligned Ni–CF fiber bundles within the PEGDA matrix and preserves their original orientation. In contrast, delayed curing may lead to fiber misalignment caused by residual resin flow, surface tension-driven spreading, and resin relaxation, ultimately compromising the structural fidelity and overall performance of the resulting composites.

The spatial distribution and orientation of conductive fillers in the as-fabricated composites were characterized using a 3D X-ray micro-CT (Xradia 610 Versa, Carl Zeiss X-ray Microscopy, Inc., Pleasanton, CA, USA), operated at 80 kV and 10 W with a voxel resolution of 5.693 μm, which is sufficient to resolve individual Ni–CF geometries. The reconstructed datasets were processed using AVIZO 2022.2 software (Thermo Fisher Scientific, Waltham, MA, USA) for three-dimensional visualization and analysis. To quantify fiber orientation, the principal axis of each Ni–CF was extracted, and the angle θ between the fiber axis and a predefined reference direction was calculated. Fibers were then statistically classified based on their orientation angles to evaluate the effects of acoustic excitation and filler concentration on alignment behavior. The electrical properties of the composites were measured using a standard two-probe method. Prior to testing, conductive silver paste (K-818, Jinshida, Zhuhai, China) was applied to both ends of the samples to minimize contact resistance. The electrical resistance was measured using a digital multimeter (DEM21, DELIXI Electric, Wenzhou, China), and the electrical conductivity σ was calculated as follows:

(7) $$\sigma =L/\left(R\cdot A\right)$$

where *L* is the sample length, *A* is the cross-sectional area, and *R* is the measured resistance. To evaluate the anisotropic conductivity induced by acoustic patterning, electrical conductivity was measured both parallel and perpendicular to the fiber alignment direction. For each experimental condition, at least six samples were tested, and the results are reported as mean ± standard deviation.

### 2.5. Mechanical Testing and Electromechanical Stability Evaluation

The mechanical properties of the as-fabricated composites were evaluated using a universal testing machine (ZQ-990B, Zhiqu Co., Dongguan, China) at room temperature. Uniaxial tensile tests were conducted at a constant strain rate of 10 mm/min until sample failure. During testing, stress–strain curves were continuously recorded, from which the Young’s modulus, ultimate tensile strength, and fracture strain were determined. For each experimental condition, at least six independent samples were tested to ensure statistical reliability.

To assess the electrical stability of the composites under mechanical deformation, cyclic bending tests were further performed. The bending strain was controlled by adjusting the curvature radius of the samples. During cyclic loading, the electrical conductivity was monitored in real time. The measured conductivity was normalized to its initial value to evaluate the stability of the acoustically induced conductive network under repeated deformation. This analysis provides insight into the reliability and durability of the conductive pathways formed by acoustic patterning.

### 2.6. Data Analysis and Statistical Methods

All experimental data are presented as mean ± standard deviation (SD). Statistical analysis was performed using one-way analysis of variance (ANOVA) followed by a post hoc *t*-test in Origin 2022 software (OriginLab Corporation, Northampton, MD, USA). Differences were considered statistically significant when *p* < 0.05.

## 3. Results and Discussion

### 3.1. Acoustic Field Characteristics of Acoustofluidic Capillary Nozzles

**Figure 2:**
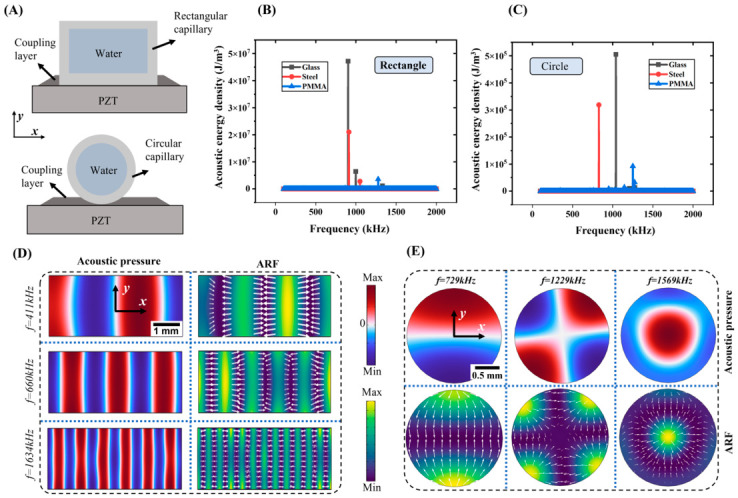
Numerical analysis of acoustic fields in capillaries with different geometries and materials. (**A**) Two-dimensional cross-sectional models of rectangular and circular acoustofluidic capillary nozzles used in simulations. (**B**) Frequency-dependent acoustic energy density of rectangular capillaries with different materials (glass, steel, and PMMA). (**C**) Frequency-dependent acoustic energy density of circular capillaries. (**D**) Acoustic pressure distributions and corresponding acoustic radiation force (ARF) fields in the rectangular capillary at selected resonance frequencies. (**E**) Acoustic pressure distributions and ARF fields in the circular capillary at different resonance modes. The white arrows in the ARF panels indicate the direction of the acoustic radiation force vectors.

Geometric structure and material properties are key factors affecting the acoustic field characteristics inside acoustofluidic capillary nozzles. Finite element simulations were conducted to clarify the effects of these two factors on acoustic energy density and acoustofluidic manipulation performance (Figure 2). The time-averaged acoustic energy density (Equation (6)) was adopted to quantify acoustic field intensity, and particle motion characteristics were analyzed based on acoustic radiation force and viscous drag force (Equations (1) and (5)). Figure 2A displays the established simulation models, including circular and rectangular capillary structures. The circular capillary possesses an inner diameter of 2 mm and a wall thickness of 0.5 mm, while the rectangular capillary has a cross-sectional size of 5 mm × 1.8 mm. Under identical excitation conditions with a peak-to-peak voltage of 20 V, the acoustofluidic responses of capillaries with different material and geometric configurations were systematically compared. To accurately capture the main acoustic characteristics while ensuring high computational efficiency, a two-dimensional cross-sectional simulation model was adopted, as particle migration and focusing behaviors are mainly governed by the standing wave distribution inside the flow channel.

As shown in Figure 2B,C, glass capillaries generally exhibit higher peak acoustic energy density than steel and PMMA capillaries over the examined frequency range. This behavior can be attributed to a combined effect of acoustic impedance matching, structural stiffness, and reduced energy dissipation, which together promote efficient acoustic coupling into the fluid domain. In addition, capillary geometry significantly modulates the spatial distribution of internal acoustic fields. Rectangular capillaries can form highly concentrated resonant peaks and regular standing wave distributions, indicating outstanding acoustic field confinement capability. By contrast, circular capillaries show more dispersed energy distribution with poor spatial aggregation of acoustic energy.

Such discrepancies in acoustic field characteristics further govern the assembly behavior of filler particles. Rectangular capillaries generate one-dimensional multi-node standing waves, where periodically distributed pressure nodes guide particles to form parallel focused lines. In circular capillaries, however, axisymmetric multi-modal acoustic fields develop, which trigger unstable particle aggregation with poor directionality, including central convergence and local enrichment. Under the experimental conditions herein, rectangular glass capillaries are more suitable than circular counterparts, owing to their tunable pressure node distribution, rapid particle focusing capability and easy assembly with PZT components.

Apart from cross-sectional shape, capillary inner diameter and outlet structure are key factors for rational nozzle design. The inner dimension determines acoustic resonance behaviors and the quantity of pressure nodes formed within the channel. A wider channel favors multi-node focusing and programmable assembly of multiple bundles, whereas an excessively narrow channel cuts down available pressure nodes and increases clogging for particle- or fiber-laden inks. Earlier works on acoustic-assisted printing adopted cylindrical tubes fitted with tapered nozzles and achieved delivery of acoustically focused microparticles into printed structures. Nonetheless, particle deposition widened after exiting the tube, an effect attributed to internal flow disturbances [52,53]. Accordingly, the present system adopts a straight rectangular capillary as the acoustic manipulation region. This design enables stable multi-node standing waves to form and minimizes geometry-induced interference prior to post-deposition curing.

### 3.2. Structural Optimization for Acoustic Energy Transmission

**Figure 3:**
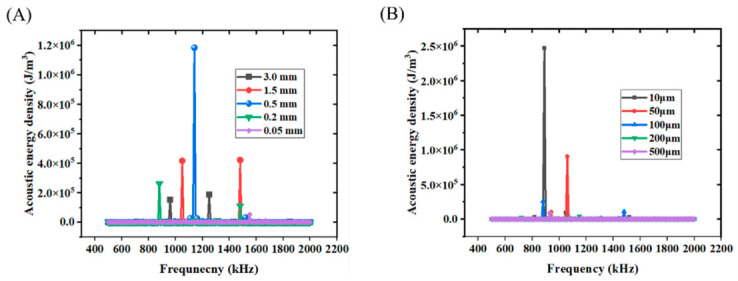
Effect of structural parameters on acoustic energy transmission in the acoustofluidic capillary nozzle. (**A**) Influence of capillary wall thickness on acoustic energy density. (**B**) Influence of coupling layer thickness on acoustic energy density.

To guide the structural design of the acoustofluidic capillary nozzle, the influence of key structural parameters on acoustic energy transmission was systematically investigated using a rectangular glass capillary as the representative configuration (Figure 3). The analysis focuses on two critical parameters: capillary wall thickness and coupling layer thickness.

The capillary wall thickness exhibits a non-monotonic effect on acoustic energy density (Figure 3A). As the wall thickness decreases from 3 mm to 0.5 mm, the acoustic energy density increases significantly, indicating enhanced energy coupling into the fluid domain. This improvement arises from reduced energy dissipation within the solid structure, allowing more acoustic energy to be transmitted into the channel. However, further reduction of wall thickness below 0.2 mm leads to a decline in acoustic energy density. This deterioration is attributed to insufficient structural stiffness, which weakens acoustic reflection at the boundary and increases energy leakage, thereby disrupting the formation of stable standing wave fields.

The coupling layer thickness exhibits a monotonic influence (Figure 3B). As the coupling layer thickness increases from 10 μm to 500 μm, the acoustic energy density decreases continuously. This behavior originates from the extended acoustic propagation path and enhanced attenuation within the coupling medium, which reduce the efficiency of energy transfer from the piezoelectric transducer to the fluid. Optimized parameter combinations enable strong and stable acoustic fields, which are essential for effective particle manipulation and microstructure control. These results indicate that both capillary wall thickness and coupling layer thickness should be carefully controlled to maximize acoustic energy coupling and ensure stable standing-wave formation within the nozzle channel.

### 3.3. Experimental Validation of Acoustofluidic Particle Focusing

**Figure 4:**
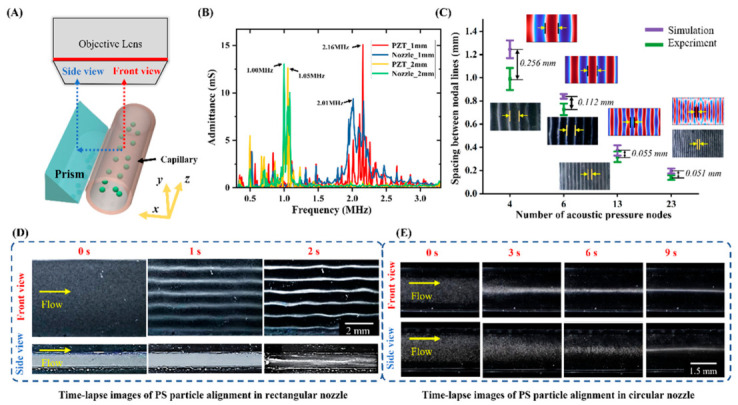
Experimental validation of particle focusing in the acoustofluidic capillary nozzle. (**A**) Schematic of the in situ observation setup. The red and blue dashed arrows indicate the front-view and side-view imaging paths, respectively. (**B**) Admittance spectra of the piezoelectric transducer and the integrated capillary nozzle system. (**C**) Comparison between experimentally observed and numerically predicted particle patterns at different excitation frequencies. The arrows indicate the spacing between adjacent particle bands or predicted pressure nodes (**D**) Time evolution of particle migration and focusing in the rectangular capillary. (**E**) Time evolution of particle migration in the circular capillary.

To experimentally validate the particle focusing capability of the acoustofluidic capillary nozzle, an in situ observation platform based on bulk acoustic wave excitation was established. As shown in Figure 4A, a right-angle prism was introduced adjacent to the nozzle, enabling simultaneous front-view and side-view observation of particle migration within the microchannel. PS microspheres with a diameter of 10 μm were used as model particles and dispersed in flowing water. Prior to particle manipulation experiments, the resonance characteristics of the acoustic nozzles were characterized using an impedance analyzer. The measured admittance spectra are shown in Figure 4B, where PZT_1 mm and PZT_2 mm represent standalone piezoelectric transducers with thicknesses of 1 mm and 2 mm, respectively, while Nozzle_1 mm and Nozzle_2 mm correspond to the integrated acoustic nozzle systems.

As shown in Figure 4B, both standalone PZT elements and integrated nozzle systems exhibit distinct resonance peaks. The resonance frequency is approximately 1 MHz for the 2 mm PZT and around 2 MHz for the 1 mm PZT, consistent with thickness-mode vibration behavior. Compared with standalone PZT elements, the integrated nozzle systems show only slight shifts in resonance frequency while maintaining consistent peak positions. This indicates that the glass capillary, coupling layer, and fluid mainly act as acoustic loading components that modulate the response without altering the dominant vibration mode. Figure 4C shows the systematically investigated particle focusing behavior of rectangular capillaries under different excitation frequencies. With increasing excitation frequency, the acoustic wavelength decreases, leading to an increase in the number of pressure nodes and a corresponding reduction in inter-node spacing. As a result, the number of particle bands increases while their spacing decreases. The good agreement between experimentally observed particle patterns and numerical predictions confirms that the standing-wave field generated inside the capillary nozzle can be predictably controlled by excitation frequency.

The dynamic process of particle focusing was further analyzed using time-resolved imaging. In rectangular capillaries (Figure 4D), under an excitation frequency of approximately 674 kHz (corresponding to ~660 kHz in simulations), particles rapidly migrated toward multiple pressure nodes and formed stable parallel structures within 2 s. In contrast, in circular capillaries (Figure 4E), under an excitation frequency of approximately 782 kHz (corresponding to ~729 kHz in simulations), particles tended to accumulate at the center or local regions, and the focusing process was significantly slower, requiring approximately 9 s. The faster focusing in rectangular capillaries can be attributed to the formation of stable one-dimensional multi-node standing wave patterns, which generate spatially uniform and directionally consistent acoustic radiation forces across the channel. This enables simultaneous particle migration toward multiple nodes. In addition, the higher acoustic energy density observed in rectangular geometries (Section 3.1) results in stronger radiation forces, further accelerating particle migration. In contrast, circular capillaries exhibit axisymmetric and multimodal acoustic fields, leading to more complex and less directed radiation force distributions, which reduces focusing efficiency. These results demonstrate that the rectangular capillary geometry provides a more reliable acoustofluidic environment for rapid and directional particle focusing, which is essential for continuous DIW-based microstructure assembly.

### 3.4. Application to Acoustic-Field-Induced Fiber Alignment in DIW

**Figure 5:**
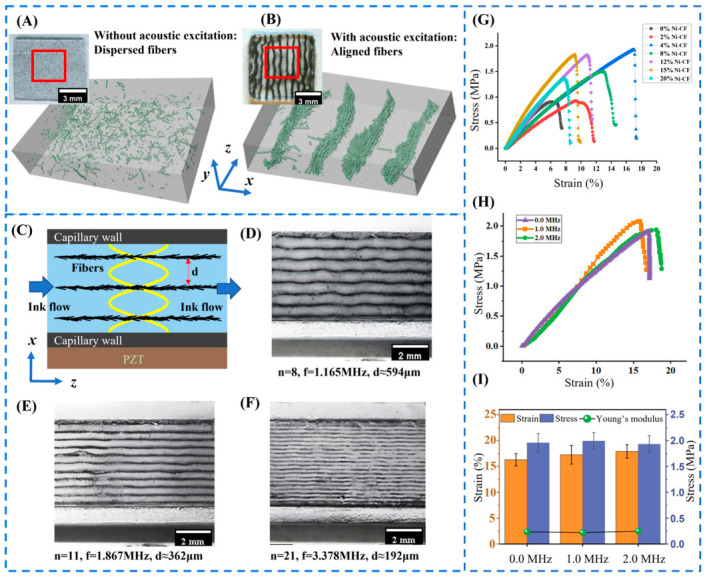
Microstructure formation and mechanical performance of Ni–CF reinforced composites fabricated using the acoustofluidic capillary nozzle. (**A**,**B**) Comparison of composites printed without and with acoustic excitation, together with corresponding three-dimensional CT reconstructions, showing randomly dispersed fibers and aligned fiber bundles, respectively. The red boxes in (**A**,**B**) indicate the representative regions selected for further visualization and analysis. (**C**) Schematic illustration of acoustic field-induced fiber alignment. The yellow curves schematically indicate the standing acoustic pressure field and pressure-node distribution. (**D**–**F**) Programmable fiber alignment patterns achieved by varying excitation frequency. (**G**) Stress–strain curves of composites with different Ni–CF contents. (**H**) Stress–strain curves of composites with and without acoustic excitation at 4 wt% Ni–CF. (**I**) Comparison of fracture strain (%, left *y*-axis), tensile strength (MPa, right *y*-axis), and Young’s modulus (MPa, right *y*-axis) under different conditions.

After confirming the acoustofluidic focusing capability of the capillary nozzle using model particles, the nozzle was further applied to in situ assembly of conductive fillers during DIW. Nickel-coated carbon fibers were selected as functional fillers to demonstrate the ability of the acoustofluidic nozzle to construct ordered microstructures within printed composites, as shown in Figure 5. The acoustic contrast factor of the Ni–CF/PEGDA-400 system was calculated using Equation (4) and the typical density and compressibility values reported in the literature [54]. It is theoretically feasible to drive Ni–CFs toward pressure nodes by acoustic radiation forces. This theoretical prediction was further supported by the experimental observations. The spatial distribution and alignment of Ni–CF within the matrix were characterized via CT reconstruction (Figure 5A,B). Without acoustic excitation, fibers are randomly dispersed due to flow-induced disturbances and inter-particle interactions during extrusion, resulting in an isotropic and disordered microstructure. In contrast, under acoustic excitation, Ni–CF fibers are driven toward pressure nodes by acoustic radiation forces, forming periodically aligned stripe-like structures. The fiber alignment is significantly enhanced, and the inter-bundle spacing agrees well with the theoretical node spacing determined by the acoustic wavelength.

The mechanism of fiber alignment is illustrated in Figure 5C. Under acoustic excitation, a standing wave field is established across the capillary cross-section, generating periodically distributed pressure nodes. Suspended Ni–CFs are driven by acoustic radiation forces to migrate laterally toward these pressure nodes, where they undergo spatially confined accumulation. Unlike spherical particles, fibrous fillers experience orientation-dependent acoustic effects. When an inclined fiber is located in a nonuniform acoustic-pressure field, the two ends of the fiber may experience different local radiation forces, generating an acoustic torque that promotes fiber rotation and reorientation (further details on force analysis can be found in Section S3 of the Supplementary Materials [55,56,57]). Meanwhile, the extrusion flow imposes hydrodynamic drag and shear-induced alignment on the suspended fibers, further guiding their orientation along the local flow direction. Therefore, the formation of aligned Ni–CF bundles results from the combined effects of acoustic-radiation-force-induced lateral translation, acoustic-torque-induced reorientation, and flow-induced hydrodynamic alignment.

Since the spacing between adjacent bundles is governed by the acoustic wavelength, tuning the excitation frequency allows precise and continuous control of the microstructural feature size. Based on this mechanism, programmable fiber alignment patterns were achieved by varying the excitation frequency (Figure 5D–F). Increasing the excitation frequency leads to a higher number of pressure nodes, resulting in more fiber bundles with reduced spacing. Specifically, as the frequency increases from 1.165 MHz to 3.378 MHz, the inter-bundle spacing decreases from approximately 594 μm to 192 μm. This demonstrates that microstructure can be continuously tuned without modifying material composition or printing parameters, highlighting the programmability of the acoustic approach.

In addition to microstructural programmability, the mechanical integrity of printed composites was evaluated to determine whether acoustic-field-induced fiber alignment introduced defects or compromised flexibility. As shown in Figure 5G, increasing Ni–CF content enhances tensile strength and stiffness due to improved load transfer through the fiber network. However, excessive filler loading reduces ductility, indicating a trade-off between reinforcement and flexibility. Among the tested compositions, 4 wt% Ni–CF exhibits the most balanced mechanical performance. Figure 5H,I compare the mechanical properties of composites fabricated with acoustic excitation and without acoustic excitation (denoted as 0.0 MHz) at 4 wt% Ni–CF. The results show that acoustic patterning does not deteriorate intrinsic mechanical properties. The fracture strain (17.92 ± 1.31%), tensile strength (1.93 ± 0.17 MPa), and Young’s modulus (0.24 ± 0.03 MPa) of acoustically patterned samples are comparable to those of non-acoustic samples (*p* > 0.05). This indicates that the formation of ordered microstructures does not introduce significant stress concentration or structural defects. Notably, the stress–strain curves of acoustically patterned samples are smoother, without abrupt stress drops prior to failure, suggesting a more uniform stress distribution during deformation. These results demonstrate that acoustic-induced fiber alignment enables programmable microstructure design while preserving mechanical integrity. This approach provides an effective strategy for achieving structure–property optimization and overcoming the trade-off between functional performance and mechanical flexibility in composite materials.

### 3.5. Enhanced Electrical Conductivity and Percolation Behavior Enabled by Acoustofluidic Microstructure Assembly

The three-dimensional spatial distribution and orientation of Ni–CF were characterized via X-ray computed tomography (CT). Based on the reconstructed datasets, fiber orientation was quantitatively analyzed using AVIZO 2022.2. Specifically, we extracted the principal axis of each fiber and calculated its angle θ relative to the y-direction, which was defined as the main fiber alignment direction. Fibers with θ < 45° were defined as aligned fibers, whereas those with θ ≥ 45° were regarded as disordered transverse fibers. Correspondingly, the aligned Ni–CF fraction refers to the proportion of fibers arranged along the y-direction, which serves as a quantitative index to evaluate the fiber arrangement effect induced by acoustic fields.

**Figure 6:**
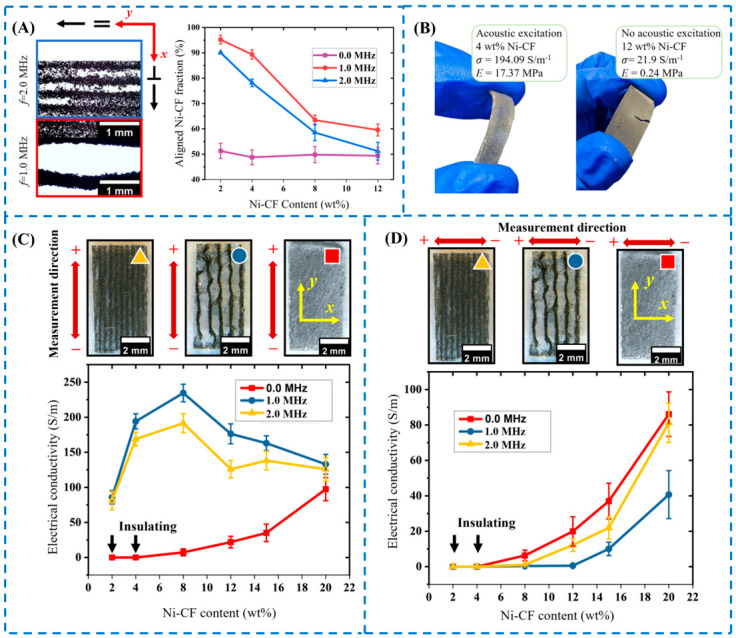
Enhanced electrical conductivity and conductive-network formation in Ni–CF reinforced composites enabled by acoustofluidic microstructure assembly. (**A**) Cross-sectional microstructures and corresponding fiber alignment ratios under different excitation conditionsThe arrow symbols indicate the measurement directions used for evaluating anisotropic conductivity, where “=” and “⊥” denote directions parallel and perpendicular to the fiber alignment, respectively. (**B**) Comparison of electrical conductivity and flexibility between acoustically assembled and randomly dispersed composites. (**C**) Electrical conductivity along the fiber alignment direction (y-direction) as a function of Ni–CF content under different excitation frequencies. (**D**) Electrical conductivity perpendicular to the alignment direction (x-direction), revealing the directional characteristics of the acoustically assembled conductive network. The colored symbols in (**C**,**D**) correspond to the samples fabricated under different excitation conditions, consistent with the legends in the conductivity plots.

As shown in Figure 6A, the aligned Ni–CF fraction varies with acoustic excitation frequency (0.0, 1.0, and 2.0 MHz) and Ni–CF content (2–12 wt%). Without acoustic excitation (i.e., 0.0 MHz), the aligned fraction remains low (~45–50%), indicating a largely random fiber distribution. In contrast, under acoustic excitation, the aligned fraction increases significantly, reaching above 90% at low filler contents, demonstrating strong acoustic-induced alignment. However, at higher filler loadings (>8 wt%), the aligned fraction decreases, which can be attributed to fiber–fiber interactions and mechanical constraints that impede effective fiber reorientation.

Figure 6B compares the electrical and mechanical performance of acoustically patterned and randomly dispersed composites. A sample with 4 wt% Ni–CF fabricated under 1.0 MHz excitation is compared with a randomly dispersed sample containing 12 wt% Ni–CF. Despite the significantly lower filler content, the acoustically patterned composite exhibits markedly higher electrical conductivity (194.09 S/m vs. 21.9 S/m) and superior flexibility. During bending, the aligned structure maintains its integrity, whereas the randomly dispersed sample shows fracture and filler detachment. In addition, the Young’s modulus is significantly reduced (from 17.37 MPa to 0.24 MPa), indicating enhanced deformability. This comparison was intended to highlight the ability of acoustic assembly to achieve superior electrical performance at substantially lower filler loading, rather than simply improving conductivity at the same filler content.

The dependence of electrical conductivity on filler content and excitation conditions was further investigated. As shown in Figure 6C, the conductivity along the fiber alignment direction (y-direction) is significantly enhanced under acoustic excitation. Notably, the percolation threshold is reduced from approximately 8 wt% (without acoustic excitation) to around 2 wt% under acoustic fields. At 8 wt% Ni–CF, the conductivity reaches 234.3 S/m, representing an approximately 32.1-fold increase compared to the non-acoustic case. This enhancement is attributed to the formation of end-to-end connected fiber networks along the pressure nodes, enabling efficient charge transport at low filler loadings. The enhancement is particularly pronounced in the low filler regime (2–8 wt%). For example, under 1.0 MHz excitation, increasing Ni–CF content from 2 wt% to 4 wt% results in a conductivity increase from 85.8 S/m to 194.1 S/m (126% improvement). However, at higher filler contents (>12 wt%), conductivity decreases, and the performance of the 2.0 MHz case becomes inferior to that of 1.0 MHz. This behavior is attributed to increased ink viscosity and flow resistance at high concentrations, which hinder effective fiber alignment. Additionally, reduced spacing between fiber bundles leads to excessive bridging and interference, disrupting continuous conductive pathways.

Figure 6D shows the conductivity perpendicular to the alignment direction (x-direction). The conductivity in this direction is significantly lower than that in the y-direction, confirming pronounced electrical anisotropy. In the low filler regime, the transverse conductivity of acoustically patterned samples is even lower than that of randomly dispersed composites due to limited lateral connectivity. As the filler content increases, inter-bundle bridging gradually enhances transverse conduction. These results demonstrate that acoustic patterning enables programmable construction of conductive networks with tunable anisotropy. By coupling acoustic field regulation with filler content optimization, it is possible to precisely control percolation behavior and achieve high electrical performance while maintaining mechanical flexibility. This strategy effectively decouples electrical performance from high filler loading, achieves synergistic optimization of electrical conductivity and mechanical flexibility, and offers a new pathway for designing lightweight and flexible conductive composites.

### 3.6. Mechanical Flexibility and Electromechanical Stability of Printed Composites

**Figure 7:**
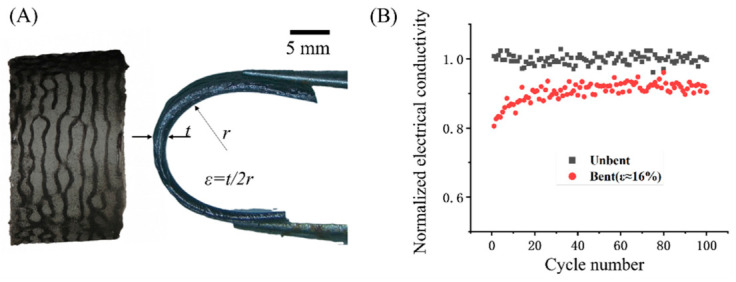
Mechanical flexibility and electromechanical stability of acoustically assembled conductive composites. (**A**) Bending configuration and strain definition, with representative images of the printed composite under different deformation states. (**B**) Normalized electrical conductivity as a function of bending cycles under unbent and bent conditions at a bending strain of approximately 16%.

To provide a preliminary evaluation of the potential applicability of acoustically assembled Ni–CF composites for flexible conductive devices, their mechanical flexibility and electrical stability under repeated bending deformation were investigated, as shown in Figure 7. Figure 7A illustrates the bending configuration and strain definition. The sample thickness is denoted as t, and the inner bending radius is defined as r. The corresponding bending strain is given by ε=t/2r. In cyclic bending tests, the samples were subjected to repeated deformation between ε=0 and ε=16% at a loading frequency of 0.5 Hz, while the electrical conductivity was monitored in real time. Figure 7B shows the evolution of normalized electrical conductivity as a function of bending cycles under both unbent and bent conditions. In the unbent state, the conductivity remains highly stable within the range of 0.98–1.02 throughout the entire cycling process, indicating excellent structural stability of the acoustically patterned conductive network. Under bending conditions (ε=16%), a slight increase in conductivity is observed during the initial cycles. This behavior can be attributed to microscale fiber rearrangement, improved inter-fiber contact, and gradual densification of the conductive network, which reduce contact resistance. As the number of cycles increases, the conductivity stabilizes within the range of 0.88–0.94, demonstrating that the conductive network maintains its integrity under repeated deformation.

The experimental results demonstrate that the acoustically assembled conductive network maintains stable electrical performance under repeated bending deformation. Combined with the improved conductivity achieved at low filler loadings, this verifies the great potential of acoustofluidic capillary nozzles in fabricating flexible conductive composites that integrate desirable electrical properties and mechanical flexibility. It should be noted that the current bending test was limited to 100 cycles at one strain level, which is not sufficient to fully assess the long-term fatigue reliability required for practical flexible conductive devices. Future work will include longer-term cyclic fatigue tests, multiple strain amplitudes, repeated electromechanical stability measurements, and device-level evaluations under practical operating conditions.

## 4. Conclusions and Future Work

In this work, an acoustofluidic capillary nozzle was developed for programmable in situ microstructure assembly during direct ink writing. By coupling a piezoelectric transducer with a commercial glass capillary, the printing nozzle was transformed into an active acoustic resonator that generates stable standing acoustic waves within the flow channel. Simulations and experiments demonstrated that rectangular glass capillaries provide a more suitable configuration under the investigated conditions, enabling stable multi-node acoustic fields, rapid particle focusing, and ordered filler assembly. The acoustofluidic capillary nozzle was further applied to the fabrication of Ni–CF reinforced conductive composites. Acoustic-field-induced fiber assembly promoted effective conductive-network formation at low filler contents, reducing the percolation threshold from 8 wt% to 2 wt% and enhancing electrical conductivity by up to 32.1-fold at identical filler contents. The printed composites also maintained good flexibility and relatively stable electromechanical performance under repeated bending. These results demonstrate that nozzle-integrated acoustofluidic assembly enables microstructure regulation beyond material-composition control, providing a simple and scalable strategy for fabricating high-performance flexible conductive composites.

Future work will focus on improving the broader applicability and scalability of the proposed acoustofluidic DIW platform. First, full three-dimensional piezoelectric–solid–fluid coupling models will be developed to further quantify axial vibration modes, inlet/outlet boundary effects, acoustic attenuation along the printing direction, and finite PZT dimensions. Second, although the present study mainly used single-filament or single-layer structures to clarify the relationship among acoustic-field distribution, filler migration, and microstructure formation, fabricating multilayer or three-dimensional structures with ordered internal filler microstructures remains an important and challenging next step. This will require coordinated optimization of path planning, interlayer bonding, curing strategy, material formulation, and acoustic-field stability during continuous printing. Third, higher-resolution characterization methods will be useful for more accurately resolving individual fiber morphology and orientation within printed composites. Finally, longer-term cyclic fatigue tests, multiple strain amplitudes, repeated electromechanical stability measurements, and device-level evaluations under practical operating conditions will be needed to further assess the reliability of acoustically assembled conductive composites for flexible conductive applications. These future efforts will help extend the proposed acoustofluidic capillary nozzle toward scalable manufacturing of multifunctional composites with programmable internal microstructures.

## Figures and Tables

**Table 1:** Material parameters for acoustic simulations.

Materials	Density (kg·m^−3^)	Speed of Sound (m·s^−1^_)_	Young’s Modulus (GPa)	Poisson’s Ratio	Acoustic Impedance (MRayl)
Steel	7850	5790	205	0.28	45.45
Glass	2203	5500	73.1	0.17	12.12
PMMA	1190	2730	3	0.40	3.25
Water (20 °C)	998	1482	—	—	1.48
Piezoelectric transducer (PZT-43)	7600	4500	65	0.3	34.20
Epoxy resin (Coupling layer)	1250	2500	1.017	0.40	3.13

## Data Availability

Data will be made available upon request.

## Supplementary Materials

The following supporting information can be downloaded at: https://www.mdpi.com/article/10.3390/mi17060744/s1, Figure S1: Configuration of the acoustofluidic DIW platform and auxiliary printing modules. (A) Schematic illustration of the three-axis motion platform and PLC-based control architecture. (B) Modular connection structure between the acoustofluidic nozzle and the Z-axis motion module; Table S1: BOM of Mechanical Motion Module.; Table S2: Parameters of PZT Materials; Figure S2: Effect of PZT material, excitation frequency, and driving voltage on the vibration response of the piezoelectric transducer. (A) Finite element model and mesh of the PZT actuator. The model had a length of 20 mm, a width of 10 mm, and a thickness of 2 mm, with the polarization direction along the thickness direction. A perfectly matched layer was introduced to reduce boundary-reflection effects. (B) Frequency-response curves of PZT-5H, PZT-43, and PZT-8 under identical voltage excitation conditions. (C) Driving voltage–vibration amplitude relationship of different PZT materials at their resonance frequencies; Figure S3: Schematic illustration of acoustic-force- and torque-induced alignment of conductive fibers in a standing bulk acoustic wave field. (A) An inclined fiber experiences asymmetric acoustic radiation forces at its two ends, generating an acoustic torque that drives rotation while promoting migration toward the pressure node. (B) Near the pressure node, the fiber reaches a more stable aligned state. The combined effects of acoustic radiation force, acoustic torque, and flow-induced hydrodynamic alignment contribute to fiber bundle formation during extrusion.

## References

[1] Agrawal P., Zhuang S., Dreher S., Mitter S., Ahmed D. (2024). SonoPrint: Acoustically Assisted Volumetric 3D Printing for Composites. Adv. Mater..

[2] Santos P.J., Gabrys P.A., Zornberg L.Z., Lee M.S., Macfarlane R.J. (2021). Macroscopic materials assembled from nanoparticle superlattices. Nature.

[3] Wang Y., Desroches G.J., Macfarlane R.J. (2021). Ordered polymer composite materials: Challenges and opportunities. Nanoscale.

[4] Pacary E., Martynoga B., Guillemot F. (2012). Crucial first steps: The transcriptional control of neuron delamination. Neuron.

[5] Xia N., Liu R., Chen W., Wang D., Sun L. (2023). Strategies for engineering neural cell alignment and their biomedical applications. Eng. Regen..

[6] Sands G., Goo S., Gerneke D., LeGrice I., Loiselle D. (2011). The collagenous microstructure of cardiac ventricular trabeculae carneae. J. Struct. Biol..

[7] Deshmukh D.V., Reichert P., Zvick J., Labouesse C., Kunzli V., Dudaryeva O., Bar-Nur O., Tibbitt M.W., Dual J. (2022). Continuous Production of Acoustically Patterned Cells Within Hydrogel Fibers for Musculoskeletal Tissue Engineering. Adv. Funct. Mater..

[8] Cyron C.J., Humphrey J.D. (2017). Growth and remodeling of load-bearing biological soft tissues. Meccanica.

[9] Rinoldi C., Costantini M., Kijeńska-Gawrońska E., Testa S., Fornetti E., Heljak M., Ćwiklińska M., Buda R., Baldi J., Cannata S. (2019). Tendon tissue engineering: Effects of mechanical and biochemical stimulation on stem cell alignment on cell-laden hydrogel yarns. Adv. Healthc. Mater..

[10] Sun J., Ye D., Zou J., Chen X., Wang Y., Yuan J., Liang H., Qu H., Binner J., Bai J. (2023). A review on additive manufacturing of ceramic matrix composites. J. Mater. Sci. Technol..

[11] Hui Z., Zhang L., Ren G., Sun G., Yu H.D., Huang W. (2023). Green flexible electronics: Natural materials, fabrication, and applications. Adv. Mater..

[12] Zheng T., Zheng X., Wang Z., Shao M., Liu X., Wang C. (2024). Manufacturing of bioinspired Bouligand structures using ultrasound assisted 3D printing. Sens. Actuators A Phys..

[13] Zimmermann E.A., Gludovatz B., Schaible E., Dave N.K., Yang W., Meyers M.A., Ritchie R.O. (2013). Mechanical adaptability of the Bouligand-type structure in natural dermal armour. Nat. Commun..

[14] Goh G.D., Yap Y.L., Agarwala S., Yeong W.Y. (2019). Recent progress in additive manufacturing of fiber reinforced polymer composite. Adv. Mater. Technol..

[15] Königsberger M., Senk V., Lukacevic M., Wimmer M., Füssl J. (2024). Micromechanics stiffness upscaling of plant fiber-reinforced composites. Compos. Part B Eng..

[16] Wei P., Leng H., Chen Q., Advincula R.C., Pentzer E.B. (2019). Reprocessable 3D-printed conductive elastomeric composite foams for strain and gas sensing. ACS Appl. Polym. Mater..

[17] Britton J., Krukiewicz K., Chandran M., Fernandez J., Poudel A., Sarasua J.R., FitzGerald U., Biggs M.J.P. (2021). A flexible strain-responsive sensor fabricated from a biocompatible electronic ink via an additive-manufacturing process. Mater. Des..

[18] Brilian A.I., Soum V., Park S., Lee S., Kim J., Kwon K., Kwon O.-S., Shin K. (2021). A simple route of printing explosive crystalized micro-patterns by using direct ink writing. Micromachines.

[19] Wang D., Wang J., Shen Z., Jiang C., Zou J., Dong L., Fang N.X., Gu G. (2023). Soft actuators and robots enabled by additive manufacturing. Annu. Rev. Control Robot. Auton. Syst..

[20] Yamagishi K., Karyappa R., Ching T., Hashimoto M. (2024). Direct ink writing of silicone elastomers to fabricate microfluidic devices and soft robots. MRS Commun..

[21] Skylar-Scott M.A., Uzel S.G., Nam L.L., Ahrens J.H., Truby R.L., Damaraju S., Lewis J.A. (2019). Biomanufacturing of organ-specific tissues with high cellular density and embedded vascular channels. Sci. Adv..

[22] Chen Y., Han P., Vandi L.-J., Dehghan-Manshadi A., Humphry J., Kent D., Stefani I., Lee P., Heitzmann M., Cooper-White J. (2019). A biocompatible thermoset polymer binder for Direct Ink Writing of porous titanium scaffolds for bone tissue engineering. Mater. Sci. Eng. C.

[23] Li C.Y., Feng C.H., Zhang L., Zhang L.J., Wang L. (2025). Direct ink writing of polymer-based materials-A review. Polym. Eng. Sci..

[24] Bernasconi R., Carrara E., Hoop M., Mushtaq F., Chen X., Nelson B.J., Pané S., Credi C., Levi M., Magagnin L. (2019). Magnetically navigable 3D printed multifunctional microdevices for environmental applications. Addit. Manuf..

[25] Plog J., Jiang Y., Pan Y., Yarin A.L. (2021). Electrostatically-assisted direct ink writing for additive manufacturing. Addit. Manuf..

[26] Yang Y., Li X., Chu M., Sun H., Jin J., Yu K., Wang Q., Zhou Q., Chen Y. (2019). Electrically assisted 3D printing of nacre-inspired structures with self-sensing capability. Sci. Adv..

[27] Compton B.G., Lewis J.A. (2014). 3D-printing of lightweight cellular composites. Adv. Mater..

[28] Hu Y. (2021). Recent progress in field-assisted additive manufacturing: Materials, methodologies, and applications. Mater. Horiz..

[29] Johnson K., Melchert D., Gianola D.S., Begley M., Ray T.R. (2021). Recent progress in acoustic field-assisted 3D-printing of functional composite materials. MRS Adv..

[30] Maramizonouz S., Jia C.F., Rahmati M., Zheng T.F., Liu Q., Torun H., Wu Q., Fu Y.Q. (2022). Acoustofluidic Patterning inside Capillary Tubes Using Standing Surface Acoustic Waves. Int. J. Mech. Sci..

[31] Wu Y., Gai J., Zhao Y., Liu Y., Liu Y. (2024). Acoustofluidic Actuation of Living Cells. Micromachines.

[32] Pelenis D., Vanagas G., Barauskas D., Dzikaras M., Mikolajūnas M., Viržonis D. (2023). Acoustic Streaming Efficiency in a Microfluidic Biosensor with an Integrated CMUT. Micromachines.

[33] Shao M., Liu X., Zheng T., Fu Y., Wang C. (2024). Acoustofluidics enables direct ink writing of vascular scaffolds with intrinsically patterned porous microstructures. Chem. Eng. J..

[34] Al Noman A., Kumar B.K., Dickens T. (2023). Field assisted additive manufacturing for polymers and metals: Materials and methods. Virtual Phys. Prototyp..

[35] Liu C., Pandit P.P., Parsons C., Khan F., Hu Y.B. (2022). Acoustic field-assisted inkjet-based additive manufacturing of carbon fiber-reinforced polydimethylsiloxane composites. J. Manuf. Process..

[36] Wadsworth P., Nelson I., Porter D.L., Raeymaekers B., Naleway S.E. (2020). Manufacturing bioinspired flexible materials using ultrasound directed self-assembly and 3D printing. Mater. Des..

[37] Friedrich L., Collino R., Ray T., Begley M. (2017). Acoustic control of microstructures during direct ink writing of two-phase materials. Sens. Actuators A Phys..

[38] Zhou Y. (2016). The Application of Ultrasound in 3D Bio-Printing. Molecules.

[39] Zhang J., Liu J., Su Q., Mao Z., Li H., Qiu Z., Peng B., Xu B. (2026). AS&SPO: A Digital Signal Phase Adjustment Method for High-precision Spool Displacement Measurement in Electro-hydraulic Control Valves. IEEE Trans. Instrum. Meas..

[40] Jonai T., Akiyama Y. (2023). Two-dimensional acoustic focusing of microparticles in a rectangular microchannel using a dual-frequency-excited single transducer. Sens. Actuators B-Chem..

[41] Bruus H. (2012). Acoustofluidics 7: The acoustic radiation force on small particles. Lab Chip.

[42] Bruus H. (2011). Acoustofluidics 1: Governing equations in microfluidics. Lab Chip.

[43] Bruus H. (2012). Acoustofluidics 2: Perturbation theory and ultrasound resonance modes. Lab Chip.

[44] King L.V. (1934). On the acoustic radiation pressure on spheres. Proc. R. Soc. London. Ser. A-Math. Phys. Sci..

[45] Gor’kov L.P. (2014). On the Forces Acting on a Small Particle in an Acoustical Field in an Ideal Fluid.

[46] Antfolk M., Muller P.B., Augustsson P., Bruus H., Laurell T. (2014). Focusing of sub-micrometer particles and bacteria enabled by two-dimensional acoustophoresis. Lab Chip.

[47] Barnkob R., Augustsson P., Laurell T., Bruus H. (2012). Acoustic radiation- and streaming-induced microparticle velocities determined by microparticle image velocimetry in an ultrasound symmetry plane. Phys. Rev. E Stat. Nonlinear Soft Matter Phys..

[48] International A. (2020). Piezoelectric Ceramics: Material Data for APC 850 (PZT-4), APC 855 (PZT-5H), and APC 841 (PZT-8).

[49] Tichý J., Erhart J., Kittinger E., Prívratská J. (2010). Fundamentals of Piezoelectric Sensorics: Mechanical, Dielectric, and Thermodynamical Properties of Piezoelectric Materials.

[50] Jaffe H. (1958). Piezoelectric ceramics. J. Am. Ceram. Soc..

[51] Tahmasebipour A., Friedrich L., Begley M., Bruus H., Meinhart C. (2020). Toward optimal acoustophoretic microparticle manipulation by exploiting asymmetry. J. Acoust. Soc. Am..

[52] Sriphutkiat Y., Zhou Y.F. (2019). Acoustic manipulation of microparticle in a cylindrical tube for 3D printing. Rapid Prototyp. J..

[53] Chow J.C., Soda K. (1972). Laminar flow in tubes with constriction. Phys. Fluids.

[54] Yu M., Zhao S., Xiao Y., Zhang R., Liu L., Chen S. (2023). High-Performance Zinc-Ion Battery Enabled by Tuning the Terminal Group and Chain Length of PEO-based Oligomers. Batter. Supercaps.

[55] Melchert D.S., Collino R.R., Ray T.R., Dolinski N.D., Friedrich L., Begley M.R., Gianola D.S. (2019). Flexible Conductive Composites with Programmed Electrical Anisotropy Using Acoustophoresis. Adv. Mater. Technol..

[56] Folorunso O., Hamam Y., Sadiku R., Ray S.S., Joseph A.G. (2019). Parametric analysis of electrical conductivity of polymer-composites. Polymers.

[57] Kyrylyuk A.V., Van Der Schoot P. (2008). Continuum percolation of carbon nanotubes in polymeric and colloidal media. Proc. Natl. Acad. Sci. USA.

