# Supplementary material for: An Acoustofluidic Capillary Nozzle for Programmable Microstructure Assembly in Direct Ink Writing of Flexible Conductive Composites
Source: Micromachines (Basel). 2026 Jun 20;17(6):744. doi: 10.3390/mi17060744 (PMC13303544; doi:10.3390/mi17060744)
Supplement: Supplementary file 1 [file micromachines-17-00744-s001.zip › micromachines-4357665-supplementary.pdf]

S1. Printing System Configuration

To support acoustofluidic direct ink writing, a modular multi-axis motion platform was designed and assembled. The platform was first modeled and assembled in SolidWorks to verify the motion path, structural dimensions, and possible mechanical interference. The overall configuration of the motion platform and control architecture is shown in Figure S1(A). The mechanical frame was constructed using 4020 and 2020 industrial aluminum profiles, and adjustable leveling feet were installed at the four corners to improve platform stability. The X- and Y-axis motions were realized using ball-screw linear stages with a travel range of 500 mm. The Z-axis motion was realized using a linear stage with a travel range of 300 mm to provide stable vertical positioning. All linear stages were driven by 42 × 35 mm² stepper motors with a shaft diameter of 5 mm, and the nominal positioning accuracy was ±2 μm. The motion control system was based on a DPK-TC55 programmable PLC controller, which supports three-axis coordinated motion and customized path planning. The stepper motors were driven by DM342 drivers, and two DC 24 V power supplies were used to independently power the PLC control unit and the motor-driving unit. The main mechanical components and specifications of the system are listed in the BOM of Table S1.

A modular nozzle-mounting structure was designed to enable stable installation and convenient replacement of acoustofluidic nozzles with different dimensions and working frequencies (Figure S1(B)). The acoustic nozzle was fixed to the connector using nylon spring clips and mounted onto the Z-axis slider using screws. This modular structure facilitates rapid nozzle replacement while maintaining mechanical stability during printing.

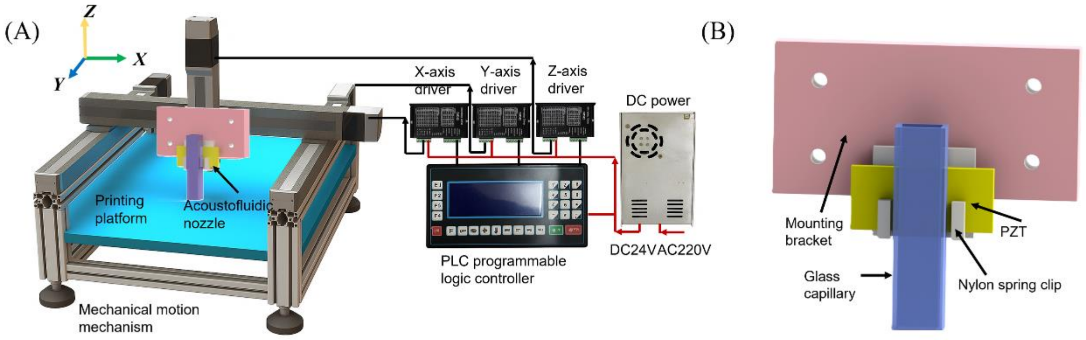

Figure S1. Configuration of the acoustofluidic DIW platform and auxiliary printing modules. (A) Schematic illustration of the three-axis motion platform and PLC-based control architecture. (B) Modular connection structure between the acoustofluidic nozzle and the Z-axis motion module.

Based on the travel ranges of the motion stages, the theoretical motion range of the platform is approximately 416 mm ×458 mm × 258 mm in the X, Y, and Z directions, respectively. The practical printable area may be slightly smaller because of the nozzle holder, tubing connection, platform clearance, and safety margin during motion. The printing resolution should be regarded as a process-dependent parameter rather than a fixed value determined only by the positioning accuracy. In addition to the nominal

positioning accuracy of the motion stages, the printed feature size is affected by the capillary outlet dimension, ink rheology, extrusion flow rate, printing speed, and post-deposition curing conditions.

Table S1. BOM of Mechanical Motion Module

Category	Item	Model/Specification	Quantity
Structural Components	4020 aluminum profile	700 mm	2
	4020 aluminum profile	660 mm	2
	4020 aluminum profile	200 mm	4
	2020 aluminum profile	500 mm	2
	Screw foot	10-80-40R	4
Transmission & Actuation Components	Stepper motor	42×35 (shaft diameter 5 mm)	4
	Ball screw linear stage	500 mm travel	3
	Ball screw linear stage	300 mm travel	1
Control & Power Supply Module	Motor driver	DM342	3
	PLC controller	DPK-TC55	1
	Power supply	DC24V	2

In this work, we primarily used single-filament and single-layer structures to systematically characterize acoustic-field-induced filler arrangement within extruded filaments. We acknowledge that fabricating complex multi-layer or 3D structures with well-ordered internal filler microstructures remains a major challenge for future research. Such fabrication requires not only acoustic-assisted filler organization, but also precise coordination of printing parameters, interlayer bonding, curing strategies, material formulations, and acoustic field stability during continuous printing to ensure reliable print quality. Further optimization of printing path planning and acoustic field stability is also necessary to achieve complex structural printing.

S2. Effects of PZT Type, Excitation Frequency and Driving Voltage on Vibration Response

To investigate the influence of piezoelectric material properties on the vibration characteristics of the transducer, three commonly used PZT materials, namely PZT-5H, PZT-43, and PZT-8, were compared. These materials exhibit distinct piezoelectric constants, dielectric properties, mechanical quality factors, and electromechanical coupling coefficients. The detailed material parameters are summarized in Table S2.

A piezoelectric–structural finite element model was established using COMSOL Multiphysics to analyze the vibration response of the PZT transducer. The modeled PZT actuator had a length of 20 mm, a width of 10 mm, and a thickness of 2 mm. The upper and lower surfaces were defined as electrode surfaces, and the polarization direction was set along the thickness direction. The side boundaries were treated as free boundaries to approximate the thickness-extension vibration mode. A perfectly matched layer was introduced outside the computational domain to reduce boundary-reflection effects. The finite element model and mesh are shown in Figure S2(A).

Table S2 Parameters of PZT Materials[1-3]

Parameter	Unit	PZT-5H	PZT-43	PZT-8
Density ρ	Kg·m^{-3}	7500	7600	7680
Sound Velocity v	m·s^{-1}	3820	4500	4530
Dielectric Constant ε_{33}	—	3400	1300	800
Piezoelectric Strain Constant d_{33}	Pc·N^{-1}	593	289	225
Electromechanical Coupling Coefficient k_{33}	—	0.75	0.48	0.36
Mechanical Quality Factor Q_m	—	65	500	1000
Mechanical Compliance s_{33}^{E}	10^{-12} m^2·N^{-1}	9.09	7.19	6.45
Relative Dielectric Loss	tanδ	0.02	0.003	0.002
Maximum Operating Temperature	℃	200	300	350

The frequency-response results are shown in Figure S2(B). All three PZT materials exhibited evident resonance peaks within the investigated frequency range, indicating that the thickness-extension vibration mode could be effectively excited under voltage actuation. Among them, PZT-5H produced the largest vibration amplitude near its resonance frequency, while PZT-8 exhibited a lower but more stable vibration response. PZT-43 showed an intermediate vibration amplitude and a relatively stable frequency response. The voltage–displacement relationship was further evaluated at the resonance frequency of each PZT material, as shown in Figure S2(C). Within the investigated voltage range, the vibration amplitude increased approximately linearly with the driving voltage, indicating that the transducers operated in a linear response regime. This result also suggests that the vibration amplitude used in the simplified acoustic-field model can be reasonably estimated from the voltage–displacement response under the corresponding experimental driving condition.

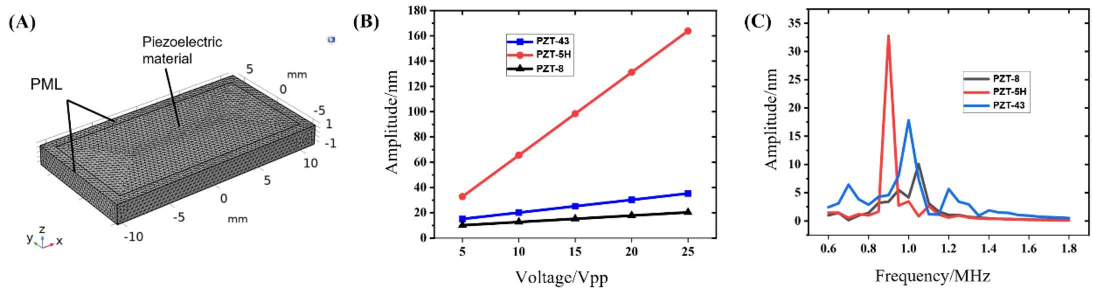

Figure S2. Effect of PZT material, excitation frequency, and driving voltage on the vibration response of the piezoelectric transducer. (A) Finite element model and mesh of the PZT actuator. The model had a length of 20 mm, a width of 10 mm, and a thickness of 2 mm, with the polarization direction along the thickness direction. A perfectly matched layer was introduced to reduce

boundary-reflection effects. (B) Frequency-response curves of PZT-5H, PZT-43, and PZT-8 under identical voltage excitation conditions. (C) Driving voltage–vibration amplitude relationship of different PZT materials at their resonance frequencies.

Based on the combined analysis of material parameters, frequency-response characteristics, and voltage–displacement behavior, PZT-43 was selected as the actuator material in this study. PZT-5H generated the largest vibration amplitude near resonance, but its lower mechanical quality factor and higher dielectric loss may reduce response stability under continuous driving or slight frequency deviation. PZT-8 exhibited better resonance stability, but its lower piezoelectric coefficient resulted in a smaller vibration output under the same excitation condition. In comparison, PZT-43 provided a more balanced performance between vibration amplitude and response stability. Therefore, the vibration amplitude of PZT-43 under the experimental driving condition was used as the harmonic displacement input in the simplified acoustic-field model.

In the acoustic-field simulations of capillary nozzles, different displacement-excitation regions were used for rectangular and circular capillaries according to their contact geometry with the planar PZT. For the rectangular capillary, the flat wall bonded to the PZT was assumed to receive effective mechanical excitation over the contact region. For the circular capillary, only a limited arc region was directly coupled to the planar PZT through the epoxy layer; therefore, the harmonic displacement was applied to approximately one-quarter of the circular wall. This boundary treatment approximates the geometry-dependent mechanical coupling between the PZT and the capillary wall while maintaining a consistent excitation basis for comparative analysis.

S3. Acoustic Radiation Force, Acoustic Torque, and Hydrodynamic Alignment of Conductive Fibers

Compared with spherical particles, the acoustic response of fibrous fillers is more complex because their motion involves not only translational migration toward pressure nodes but also rotational reorientation[4]. In a standing bulk acoustic wave field, a conductive fiber can be approximated as a slender rod subjected to a distributed acoustic radiation force. Because the two ends of an inclined fiber are generally located at different acoustic-pressure gradients, the local acoustic radiation forces acting on the two ends are not identical， as shown in Figure S3. This asymmetric force distribution generates an acoustic torque around the fiber center, which drives fiber rotation and reorientation toward a more stable configuration near the pressure node. The acoustic radiation force (F_{rod}) and acoustic torque (τ_{rod}) acting on a slender rod can be expressed as follows[5,6]:

$$F_{rod} = -d^2 E_{ac} \sin(2kx)\, 2\phi |\sin\theta| \cos^{-1}\theta \sin(kl\cos\theta) + \pi k d \phi_{end} \cos\theta \cos(kl\cos\theta) \quad (1)$$

$$\tau_{rod} = \frac{d^2}{k} E_0 \phi \tan^2\theta \cos(2kx)[\,(-kl\cos\theta)\cos(kl\cos\theta) + \sin(kl\cos\theta)] +$$

$$\left(\frac{\pi}{2}\right) kld^3 E_{ac}\phi_{end} \sin\theta\,|\cos\theta|\cos(2kx)\sin(kl\cos\theta) \quad (2)$$

where d is the fiber diameter, l is the fiber length, θ is the angle between the fiber and the x-axis, ϕ is the acoustic contrast factor, $E_{ac}$is the acoustic energy density, and ϕ_{end} is the end-effect enhancement factor.

Figure S3 schematically illustrates the force and torque acting on a conductive fiber in a standing acoustic wave field. In the initial state, randomly distributed fibers experience acoustic radiation forces that drive their translational migration toward the pressure nodes. When the two ends of an inclined fiber are located at different distances from the pressure node, the resulting asymmetric radiation forces produce a nonzero acoustic torque, leading to fiber rotation and orientation adjustment. As the fiber approaches the pressure node, the local acoustic-pressure gradient decreases and the translational force becomes weaker, while the torque-assisted reorientation helps the fiber reach a more stable aligned state.

In addition to acoustic radiation force and acoustic torque, hydrodynamic effects during extrusion also contribute to fiber alignment and bundle formation. The flow field inside the capillary nozzle imposes viscous drag and shear-induced rotational effects on the suspended fibers. These hydrodynamic effects tend to align fibers along the local flow direction during extrusion, whereas the acoustic radiation force concentrates fibers laterally toward the pressure nodes. Therefore, the final bundle formation results from the combined action of acoustic-field-induced lateral translation, acoustic-torque-induced reorientation, and flow-induced hydrodynamic alignment. At higher fiber concentrations, fiber–fiber interactions further influence the alignment process. Local fiber accumulation near the pressure nodes increases the probability of contact, steric hindrance, and partial entanglement. Some fibers may not fully rotate into the local stable orientation and may bridge adjacent focused bundles. These bridging structures can provide additional transverse conductive connections, contributing to the anisotropic conductive behavior of the printed composites.

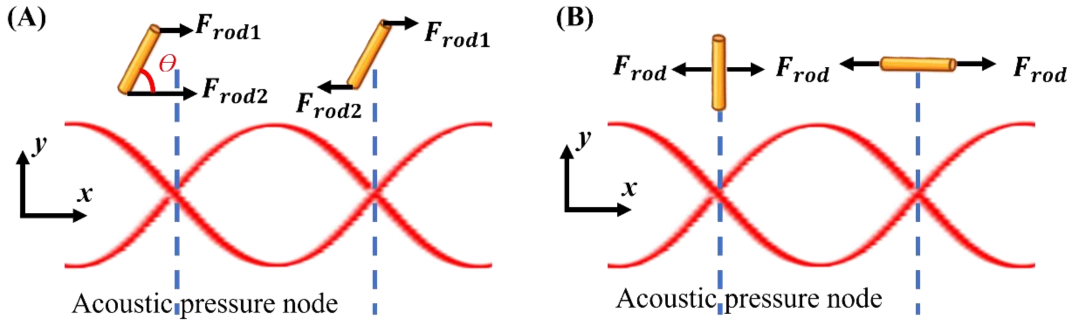

Figure S3. Schematic illustration of acoustic-force- and torque-induced alignment of conductive fibers in a standing bulk acoustic wave field. (A) An inclined fiber experiences asymmetric acoustic radiation forces at its two ends, generating an acoustic torque that drives rotation while promoting migration toward the pressure node. (B) Near the pressure node, the fiber reaches a more stable aligned state. The combined effects of acoustic radiation force, acoustic torque, and flow-induced hydrodynamic alignment contribute to fiber bundle formation during extrusion.

References

1. International, A. *Piezoelectric Ceramics: Material Data for APC 850 (PZT-4), APC 855 (PZT-5H), and APC 841 (PZT-8)*; APC International: Mackeyville, 2020.
2. Tichý, J.; Erhart, J.; Kittinger, E.; Prívratská, J. *Fundamentals of piezoelectric sensorics: mechanical, dielectric, and thermodynamical properties of piezoelectric materials*; Springer Science & Business Media: Berlin, 2010.
3. Jaffe, H. Piezoelectric ceramics. *Journal of the American Ceramic Society* **1958**, *41*, 494-498, doi:10.1111/j.1151-2916.1958.tb12903.x.
4. Melchert, D.S.; Collino, R.R.; Ray, T.R.; Dolinski, N.D.; Friedrich, L.; Begley, M.R.; Gianola, D.S. Flexible Conductive Composites with Programmed Electrical Anisotropy Using Acoustophoresis. *Advanced Materials Technologies* **2019**, *4*, 1900586, doi:10.1002/admt.201900586.
5. Folorunso, O.; Hamam, Y.; Sadiku, R.; Ray, S.S.; Joseph, A.G. Parametric analysis of electrical conductivity of polymer-composites. *Polymers* **2019**, *11*, 1250, doi:10.3390/polym11081250
6. Kyrylyuk, A.V.; Van Der Schoot, P. Continuum percolation of carbon nanotubes in polymeric and colloidal media. *Proceedings of the National Academy of Sciences* **2008**, *105*, 8221-8226, doi:10.1073/pnas.0711449105.
